# Expansion of our editorial board

**DOI:** 10.1111/iwj.70010

**Published:** 2024-08-18

**Authors:** Keith Harding, Douglas Queen

**Affiliations:** ^1^ International Wound Journal Oxford United Kingdom

Like many journals, the IWJ has seen a significant increase in submissions, with some of it being our natural growth as an important resource for those involved in wounds. However, some of the increase is related to “papermill” type activity. This has challenged our submission and review process. To manage this, we are implementing significant back‐end processes to identify and reject such content. We are also creating the largest single editorial board for any wound care journal, to strengthen our review process to enhance its effectiveness. This has become necessary to ensure the continued quality of our journal, especially considering the threat that “papermill type” activity may have on our overall subject area. This is a global challenge to the world of academic and clinical research. It is something that the IWJ does not take lightly and we are working diligently in playing our part to minimize its impact in the world of wound care.

We are pleased to announce the expansion of our editorial board (Figure 1). Our founding members who remain on our board become our senior editorial advisers. We would be remis, however, if we did not thank our retiring board members for their service and remember those who are no longer with us (Table 1).

**FIGURE 1:**
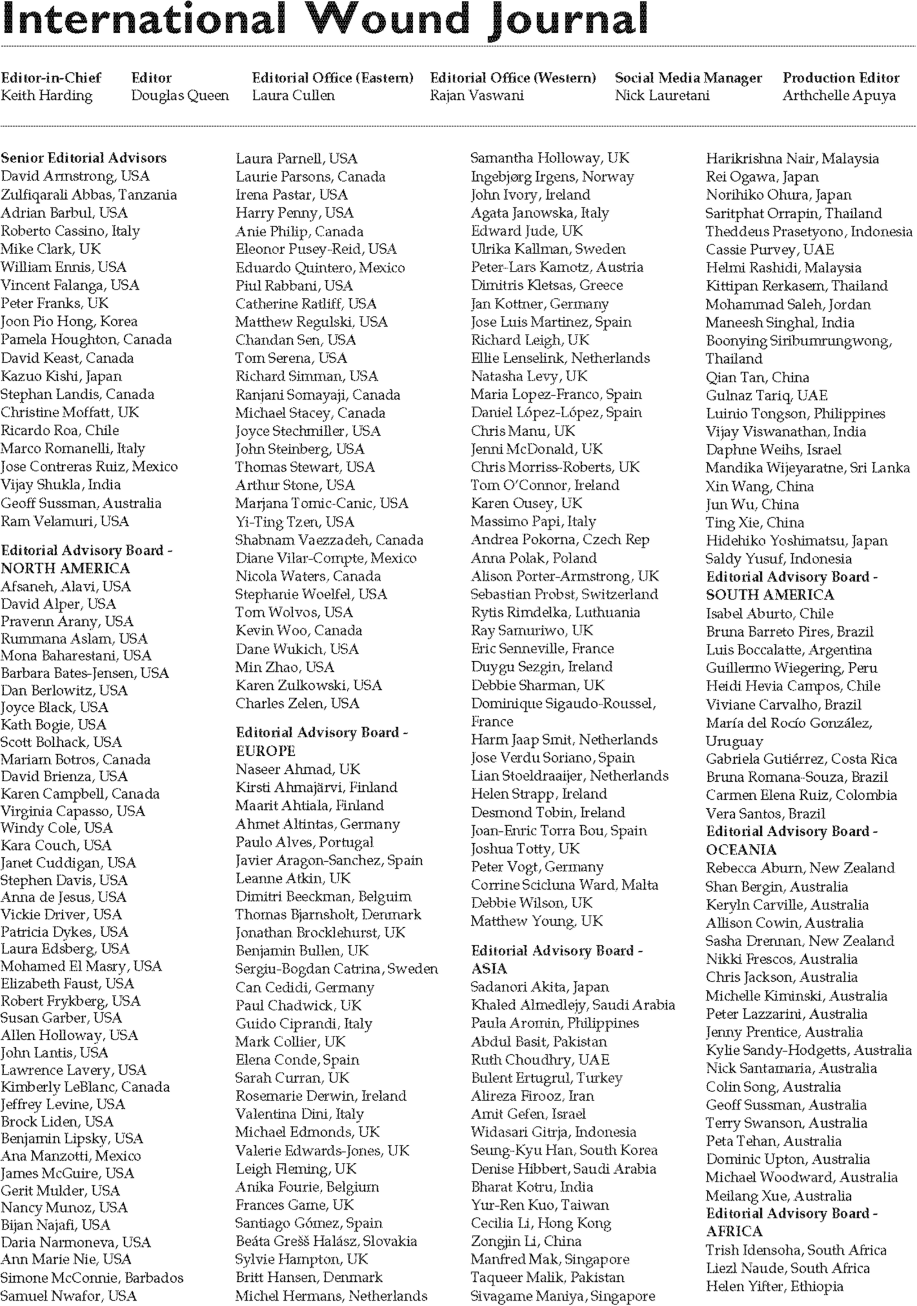
Our newly expanded editorial board (2024).

**TABLE 1:** Our retiring and departed founding members.

Retiring Editorial Members	Patricia Price, UK David Leaper, UK Kyoichi Matsuzaki, Japan Heather Orsted, Canada Bishara Atiyeh, Lebanon Paul Banwell, UK Suzie Calne, UK Diane Cooper, USA Ian Darby, Australia Jeff Davidson, USA Sebastian Debus, Germany Evonne Fowler, USA Xiabong Fu, China Finn Gottrup, Denmark Satsue Hagisawa, Japan Eva‐Lisa Heinrichs, UK Dirk Hollander, Germany Kent Jonsson, Zimbabwe	Diane Krasner, USA Luis Fernando Lira, Mexico Courtney Lyder, USA Mario Marazzi, Italy Sylvie Meaume, France Joe McCulloch, USA Yvette Moulin, Canada Manoj Pandey, India Nancy Parslow, Canada Hermelinda Pedrosa, Brazil Elaine Pina, Portugal Elia Ricci, Italy George Rodeheaver, USA Hiromi Sanada, Japan Gary Sibbald, Canada Mark Tang, Singapore Luc Teot, France Cath Vowden, UK Peter Vowden, UK
Editorial Members We Lost	Gregory Schultz, USA; Hugo Partsch, Austria; George Cherry, UK	

This is the first editorial in a series where we will introduce our newly expanded editorial board. We have created the largest most internationally diverse board to greatly increase the capabilities and expertise of the journal as it moves to its third decade of life. From Figure 1, you can see we have expanded our board to include over 240 plus members. These individuals are diverse geographically, diverse in speciality, diverse in experience and gender balanced.

The first stage of our board expansion was to establish those who wished to continue and to use their experience in creating our Senior Editorial Advisors. Here you will be introduced to our founding board members who decided to continue and become the senior advisors. We are privileged and honoured to have such high‐calibre members continuing on our board.

## 1 SENIOR EDITORIAL ADVISORS

Please meet the Senior Editorial Advisors for the International Wound Journal:

**Table:** 

**Dr Zulfiqarali Abbas, Tanzania**	
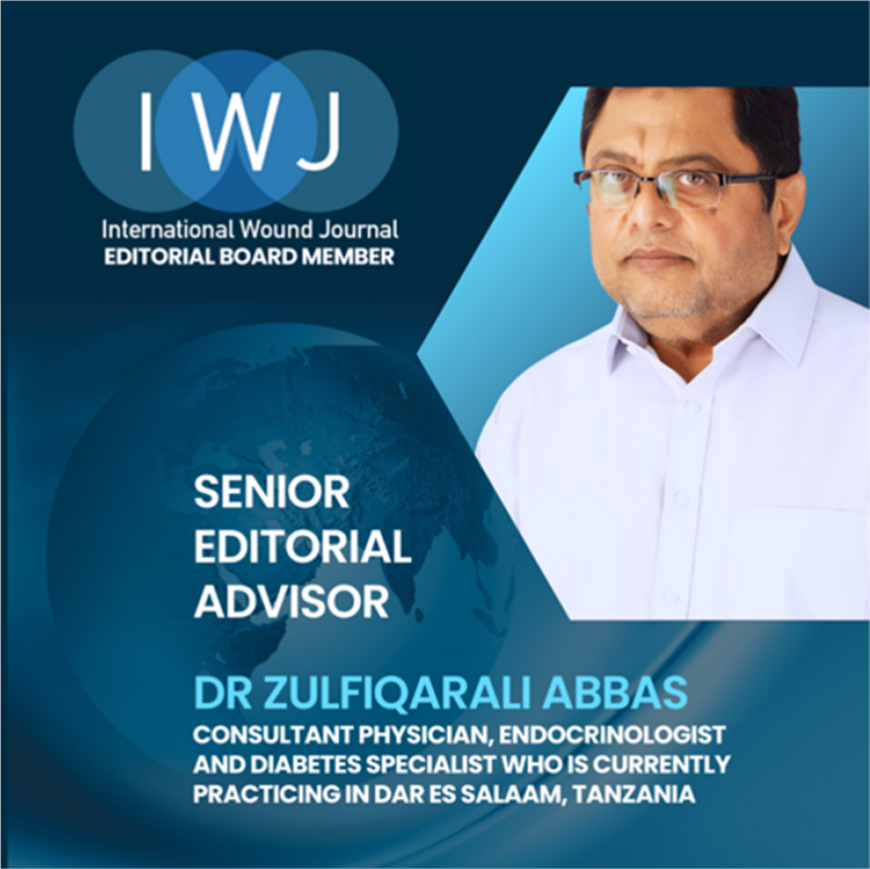	Dr. Abbas is a consultant physician, endocrinologist and diabetes specialist who is currently practicing in Dar es Salaam, Tanzania. Since 1992, he has been Honorary Physician and Research Fellow in the Department of Internal Medicine at Muhimbili University of Health and Allied Sciences (MUHAS). His clinical and academic commitments have included running the diabetes clinic at Muhimbili National Hospital (MNH) and Abbas Medical Centre and teaching activities at MUHAS in Dar es Salaam. He completed postgraduate medical training in the United Kingdom and Tanzania and was awarded an International Fellowship in epidemiology by the CDC in Atlanta, USA. Last year he was awarded FRCP honours from the Royal College of Physicians and Surgeons of Glasgow. Dr. Abbas is the current chair of the Pan‐Africa Diabetic Foot Study Group and, at present, sits on the editorial board of several international peer‐reviewed journals. Dr. Abbas has written extensively and has approximately 50 publications in peer‐reviewed journals. As an invited speaker, Dr. Abbas has given over 100 state‐of‐the‐art lectures at national and international conferences, and has written several book chapters, including a pocketbook on diabetic foot for healthcare workers. Dr. Abbas has led the country in highlighting the utility and rationale use of microbiology resources in the management of infections in persons with diabetes.
**Professor David G. Armstrong, USA**	
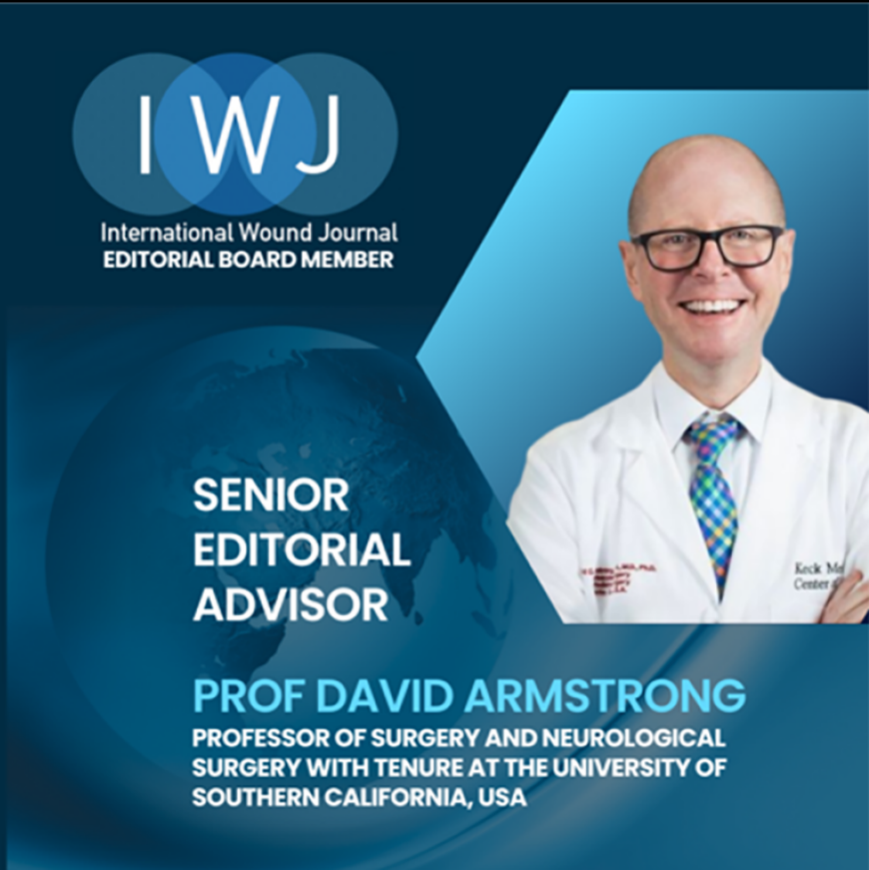	Dr. Armstrong is Professor of Surgery and Neurological Surgery with Tenure at the University of Southern California. Dr. Armstrong holds a Master of Science in Tissue Repair and Wound Healing from the University of Wales College of Medicine and a PhD from the University of Manchester College of Medicine, where he was appointed Visiting Professor of Medicine. He is founder and co‐Director of the Southwestern Academic Limb Salvage Alliance (SALSA). Dr. Armstrong has produced more than 675 peer‐reviewed research papers in dozens of scholarly medical journals as well as over 110 books or book chapters. He is founding co‐Editor of the American Diabetes Association's (ADA) Clinical Care of the Diabetic Foot. Armstrong is Director of USC's National Science Foundation (NSF) funded Center to Stream Healthcare in Place (C2SHiP), which places him at the nexus of the merger of consumer electronics, wearables and medical devices in an effort to maximize hospital‐free and activity‐rich days. In 2008, he was the 25th and youngest ever member elected to the Podiatric Medicine Hall of Fame. He was also the first podiatric surgeon to be selected as President of Faculty at Keck School of Medicine of USC. Dr. Armstrong is the founder and co‐chair of the International Diabetic Foot Conference (DF‐Con), the largest annual international symposium on the diabetic foot in the world. He is also the Founding President of the American Limb Preservation Society (ALPS). ORCID: (https://orcid.org/0000‐0003‐1887‐9175)
**Professor Adrian Barbul, USA**	
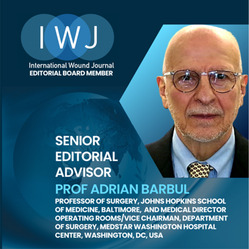	Dr. Barbul is the Professor of Surgery at Johns Hopkins School of Medicine, Baltimore, MD and Medical Director of Operating Rooms/Vice Chairman, Department of Surgery, Medstar Washington Hospital Center, Washington, DC. Dr. Barbul's research is focused on surgical nutrition, amino acid metabolism and research pertaining to the treatment of wound healing. He has most recently published articles including, Effect of a Specialized Amino Acid Mixture on Human Collagen Deposition, Annals of Surgery, the Role of Nitric Oxide in Repair Processes, American Journal of Surgery, and Excessive Nitric Oxide Impairs Wound Collagen Accumulation, Journal of Surgical Research. Dr. Barbul received his undergraduate degree from City College of New York, New York, NY and his medical degree from the School of General Medicine, Bucharest, Romania. Dr. Barbul is certified by the American Board of Surgery and is a fellow of the American College of Surgeons. For his contributions to wound healing, he has been presented with the Lifetime Achievement Award by the Wound Healing Society of America.
**Professor Roberto Cassino, Italy**	
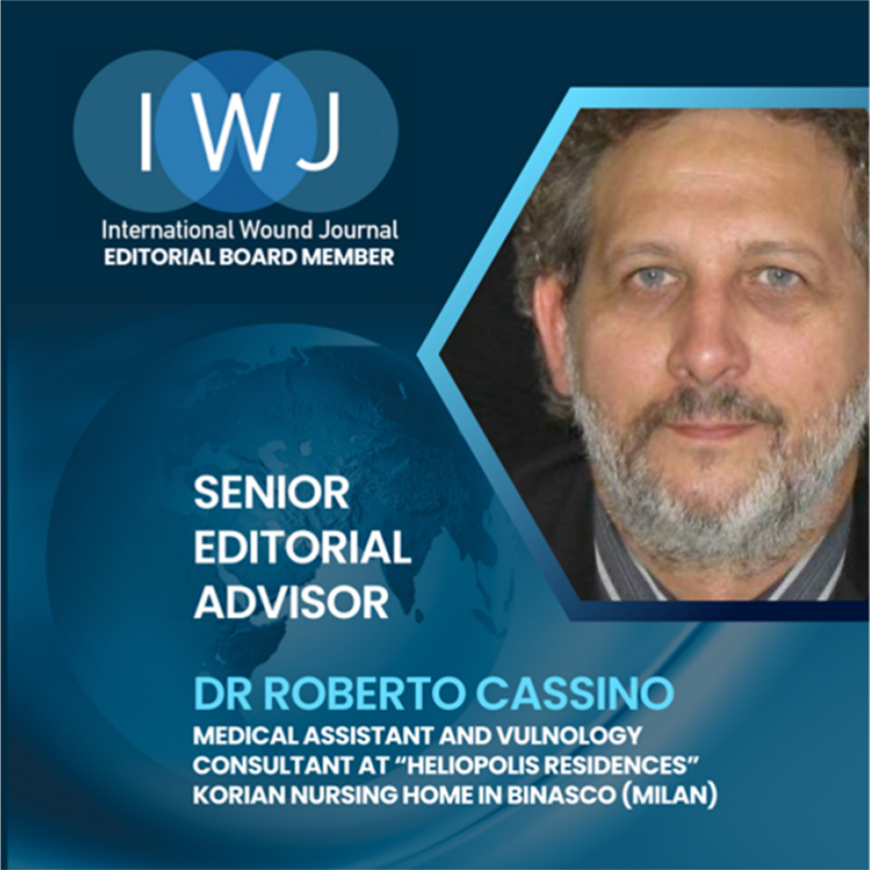	Prof. Roberto Cassino is a graduate in Medicine and Surgery at the University of Turin, specialized in geriatrics and gerontology, expert in vulnology and diabetic foot. He is a lecturer in the Masters of Vulnology at the University of Turin. Prof Cassino has worked in several Italian hospitals including “Camillo Golgi” Geriatric Institute in Abbiategrasso (Milan); San Luca Clinic (Critical Wounds Unit) in Pecetto Torinese (Turin); ICCS – “Città Studi” Clinical Institute in Milan (Interdepartmental Center for the Treatment of Diabetic Foot—Center of Vulnology) and Zucchi Clinical Institutes—Center of Vulnology, Monza. Currently, he is the Medical Assistant and Vulnology Consultant at “Heliopolis Residences” Korian Nursing Home in Binasco (Milan). Lecturer in geriatrics and vulnology in over 290 courses from 1993 to today. He is a member of the following associations: AIUC Italian Association of Cutaneous Ulcers since 1999 EWMA European Wound Management Association since 2008 EPUAP European Pressure Ulcer Advisory Panel since 2008 Director of the series “Vulnologia Pratica: per fare e saper fare…” edited by Minerva Medica and co‐author of many books of vulnology. Author of over 490 publications in vulnology. Winner of 5 prizes for best congress presentation (1998): at EWMA (1998, 2000, 2007), AIUC (2008) and WASET (2017). Winner of the Bronze Medal at JWC Olympics at the World Union of Wound Healing Societies conference 2020/2022, Abu Dhabi (UAE). Best Oral Presentation Award at AIUC conferences (2021 and 2023).
**Professor Mike Clark, UK**	
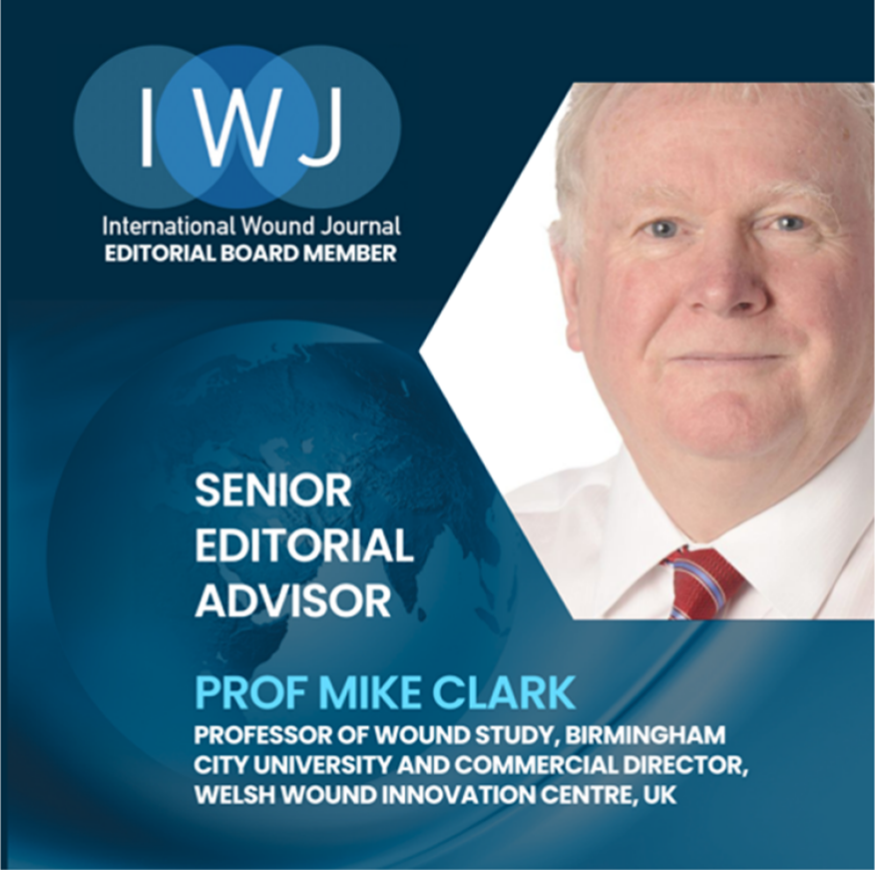	Michael Clark is Commercial Director, Welsh Wound Innovation Centre. A zoologist by training, Professor Clark has worked in tissue viability and wound healing since 1980. He is Professor of Wound Study, Birmingham City University supporting their Wound Healing Practice Development Unit 1 day a week. Between 2003 and 2009, he was a member of the Wound Healing Research Unit, Cardiff University and had responsibility for physical measurement research within the WHRU. From 2009 to 2013, Professor Clark managed the Welsh Wound Network while also acting as an independent consultant. Professor Clark was Chief Executive of the Lindsay Leg Club Foundation and has been President of the European Pressure Ulcer Advisory Panel.
**Professor William Ennis, USA**	
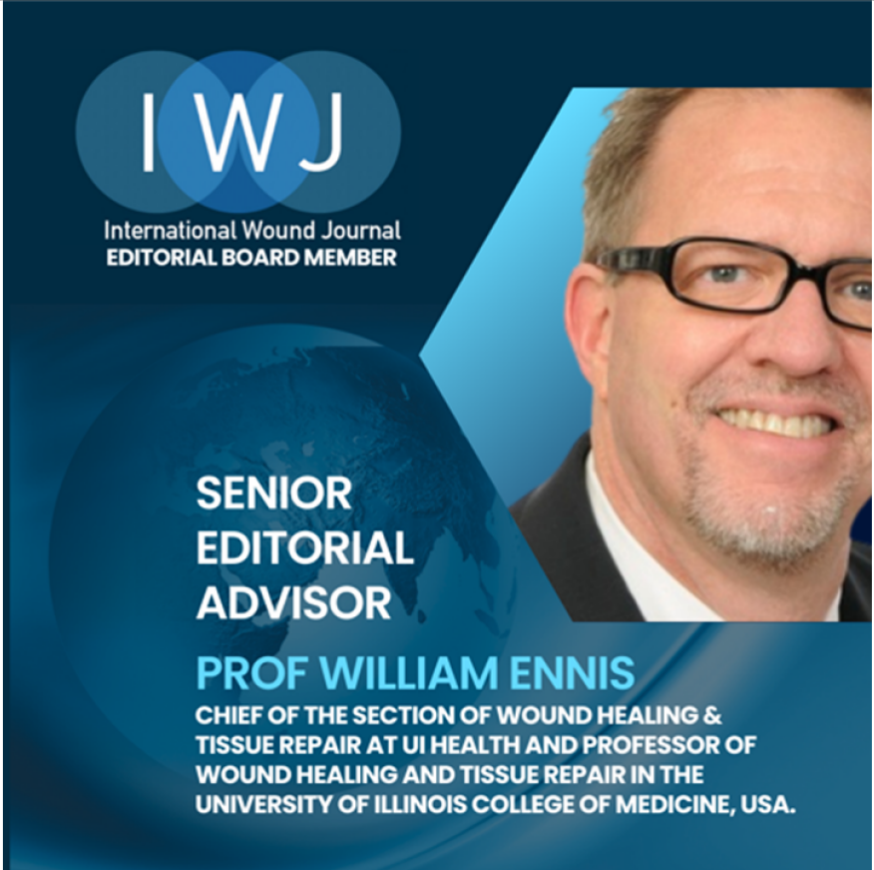	Dr. William Ennis is the chief of the Section of Wound Healing & Tissue Repair at UI Health. Dr. Ennis specializes in wound care, diabetic foot care, lower extremity wounds, pressure ulcers, Apligraf therapy and venous ulcers, and his current research interests include microcirculation, regenerative tissue mechanisms and healing outcomes. Dr. Ennis serves as the President of the American College of Wound Healing and Tissue Repair, a non‐profit organization that brings wound care to the level of a formal medical specialty, and he founded the first wound healing and tissue repair fellowship in the United States. He is the Catherine and Francis Burzik Professor of Wound Healing and Tissue Repair in the University of Illinois College of Medicine. ORCID: (https://orcid.org/0000‐0002‐5788‐9854)
**Professor Vincent Falanga, USA**	
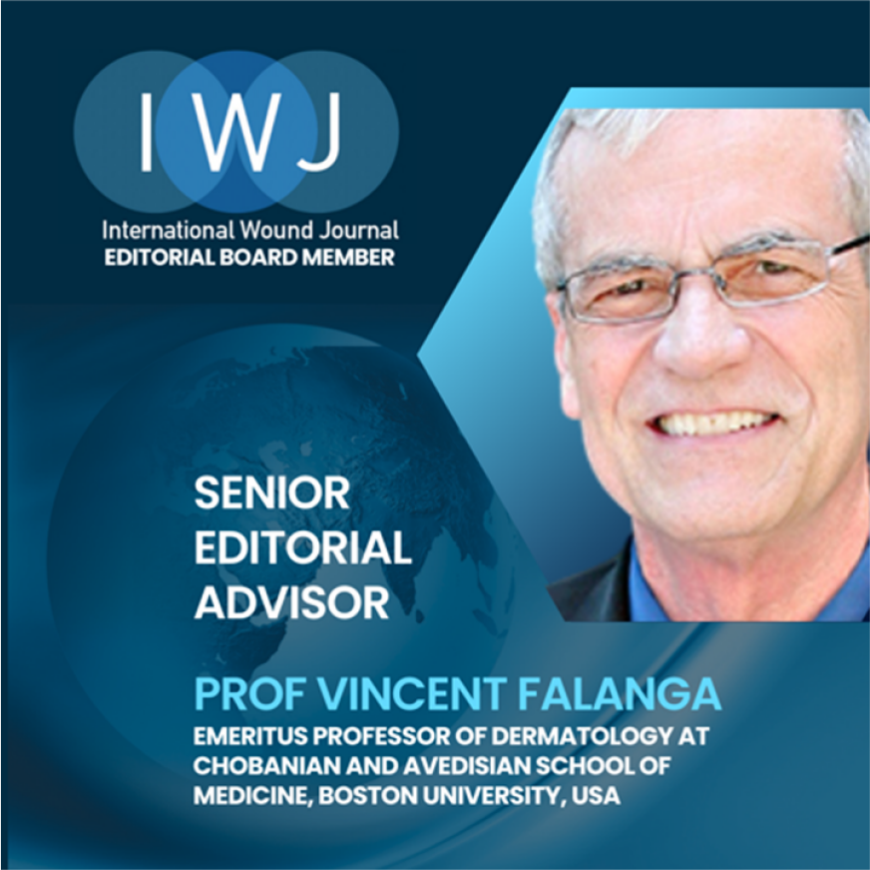	Dr. Vincent Falanga is Emeritus Professor of Dermatology at Chobanian and Avedisian School of Medicine. A dedicated researcher, clinician, and educator, Dr. Vincent Falanga has had a long‐standing relationship with the Boston University community. For the past 26 years, Dr. Falanga has been a Professor Biochemistry at the Chobanian and Avedisian School of Medicine (BUSM). In addition, he served as the Assistant Dean of Clinical and Faculty Affairs, as well as the Director of the Boston University Medical Students Ambulatory Medical Clerkship at Roger Williams Medical Center (RWMC), a research and clinical affiliate of the BUSM. From 1998 to 2013, he was the Chairman and Program Director of the Department of Dermatology at RWMC, a major affiliate of Boston University. At that institution, he was also president of the multispecialty practice group. Prior to that, Dr. Falanga held several academic appointments, including an initial one at the University of Pittsburgh School of Medicine, and then as Professor of Dermatology and Medicine at the Miller School of Medicine at the University of Miami. A graduate of Harvard Medical School, Dr. Falanga received training in Internal Medicine and Dermatology at the University of Miami and the University of Pennsylvania, respectively. He is Board certified in both Medicine and Dermatology. In 2004, he was the President of the Wound Healing Society, the premier research organization in the world dedicated to wound healing. Overall and throughout his scientific career, Dr. Falanga has received more than $45 million in research funding, mostly from the National Institutes of Health (NIH). ORCID: (https://orcid.org/0000‐0003‐2292‐2017)
**Professor Peter Franks, UK**	
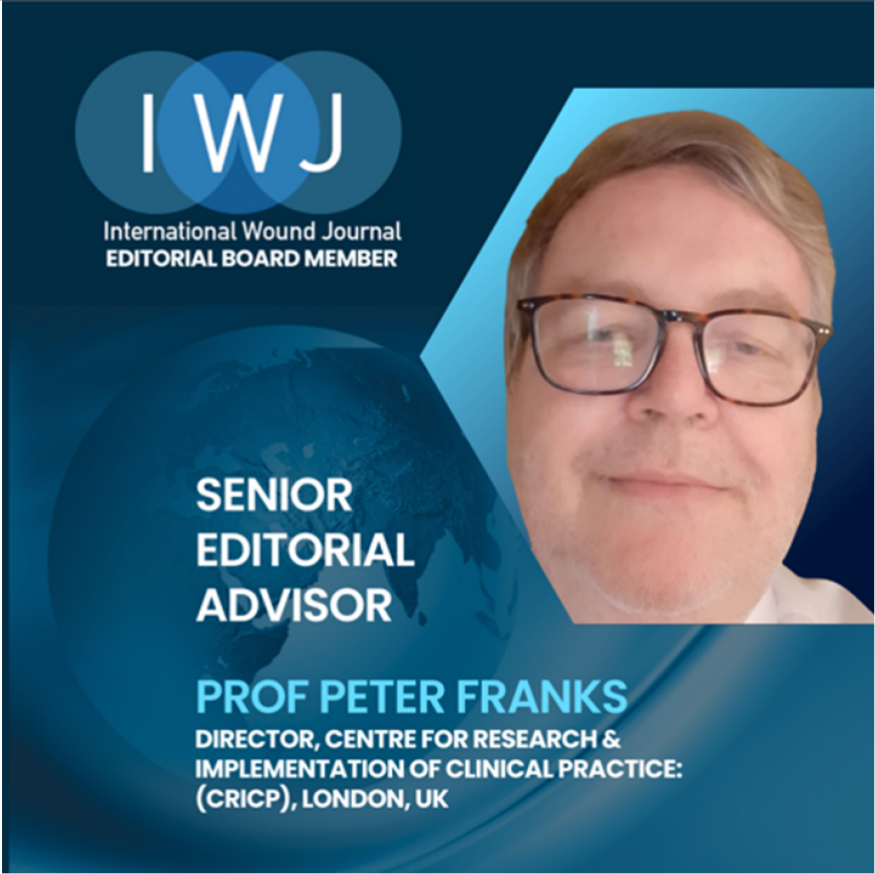	Director, CRICP—Centre for Research in Schemes and Policies—was established in January 1994 as an independent self‐funding research and education unit. The aims of the Centre are to establish areas of need and develop models of care to overcome these needs. We have successfully implemented the community leg ulcer system developed in Riverside to other trusts throughout the United Kingdom and internationally. The Centre has established links with academic and clinical groups including Portugal, United States, Japan, Canada, France and Denmark. Honorary Board Member of the International Lymphoedema Framework. Authored 146 original articles in peer‐reviewed journal. Past President of EWMA (2005‐2007) and served on the EWMA Council and its scientific committee (1997–2008). ORCID: (https://orcid.org/0000‐0002‐9782‐0181)
**Professor Joon Pio Hong, South Korea**	
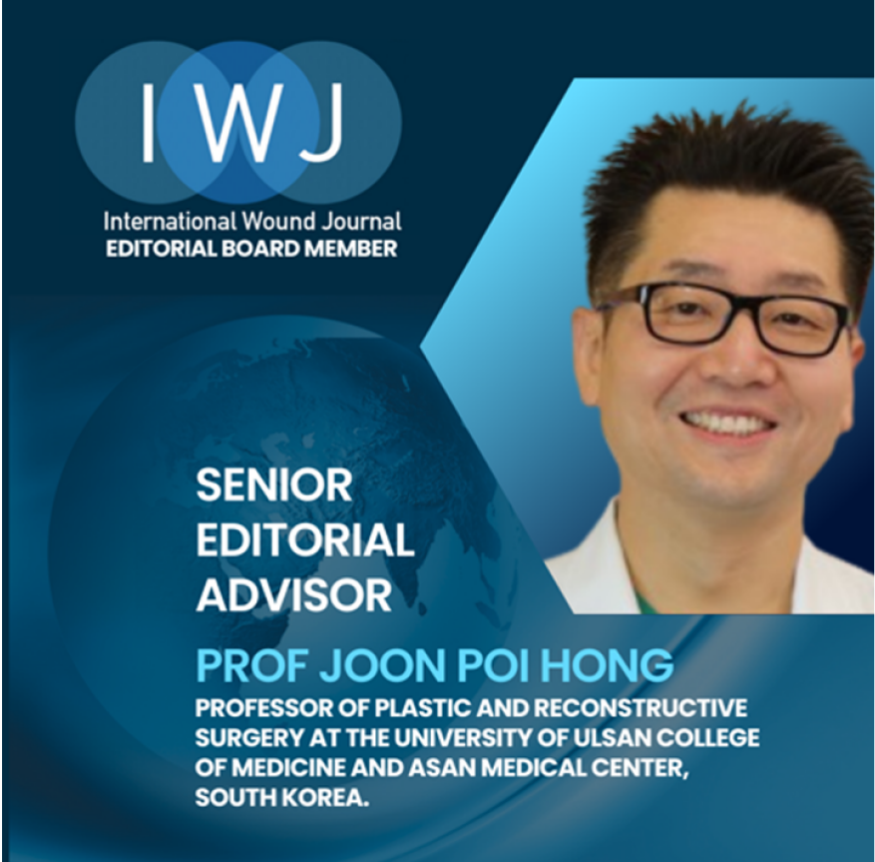	**Joon Pio Hong, M.D., Ph.D., M.B.A.** is Professor of Plastic and Reconstructive Surgery at the University of Ulsan College of Medicine and Asan Medical Center. He has board certification in trauma, hand along with plastic surgery. He received his BS degree from the Yonsei University of College of Medicine and his MS degree in medicine and PhD degree from the Graduate School of Yonsei University. He received his M.B.A. on medical management from University of Southern California at Marshall School of Business. He is an active member of a number of professional associations such as the American Society of Plastic Surgery, World Society of Reconstructive Microsurgery, and Korean Society of Plastic Surgery. His major work has been research and clinical practice in wound healing, diabetic foot reconstruction and microsurgery. He is on the editorial board for numerous journals including Plastic and Reconstructive Surgery, International Wound Journal, Journal of Reconstructive Microsurgery, Journal of Plastic and Reconstructive Surgery and more. He has been invited in over 80 countries to present his work and is visiting professor for more than 20 institutions. He has over 160 publications in this field of practice with 24 book chapters including the Neligan Plastic surgery. He was awarded the “Godina Travelling Fellowship” from the 2015 American Society of Reconstructive Microsurgery. ORCID: (https://orcid.org/0000‐0002‐6208‐9704)
**Professor Pamela Houghton, Canada**	
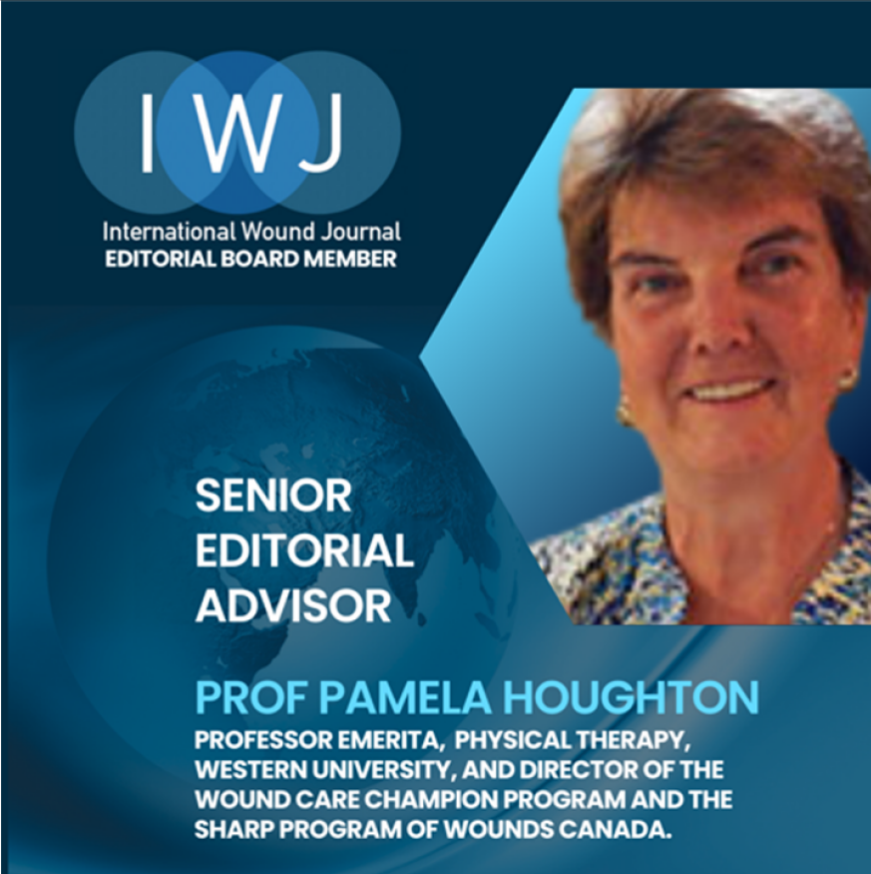	Dr. Pamela E. Houghton is Professor Emerita from the School of Physical Therapy at Western University, London, Canada. In 2007, she launched the Master of Clinical Science program in the field of wound healing. This is the only graduate programme in Canada that provides specialized training for healthcare professionals treating people with chronic wounds using distance education methods. Dr. Houghton recently retired after leading an active research program at Western that was dedicated to evaluating advanced wound treatments such as electrical stimulation for the treatment of people with chronic wounds. Pamela was a licensed Physical Therapist and member of the Canadian Physiotherapy Association for over 30 years. Dr Houghton is currently the Director of the Wound Care Champion Program and the SHARP program of Wounds Canada.
**Professor David Keast, Canada**	
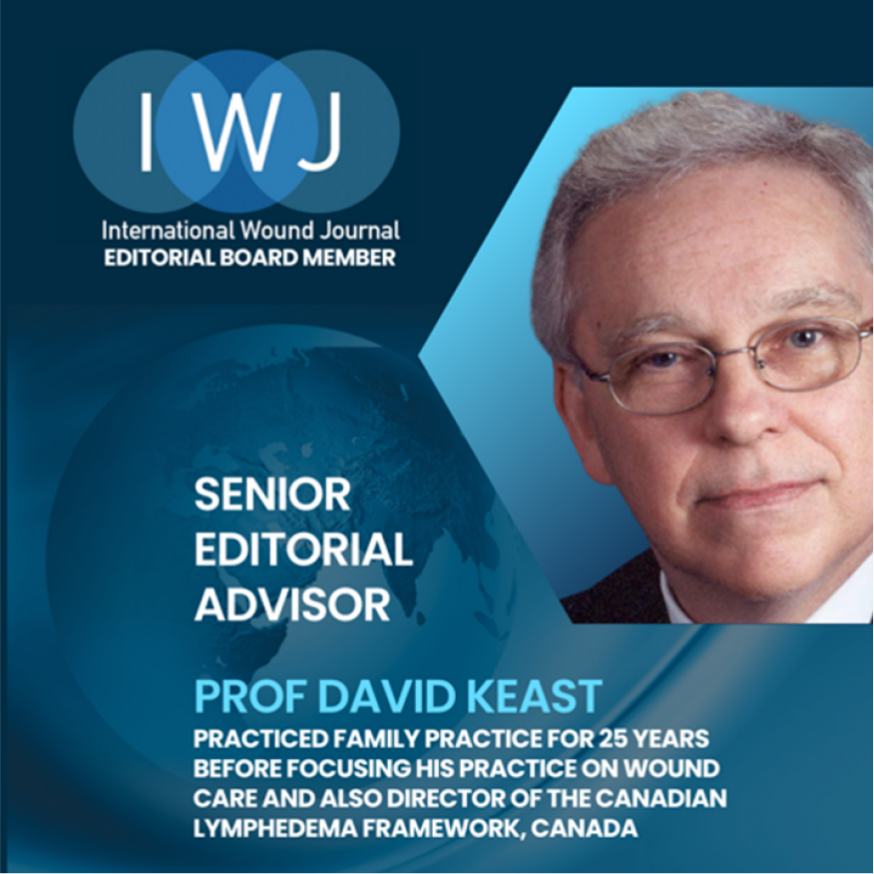	Dr. Keast is a native of London Ontario, graduating from Western University with a master's degree in chemistry. He taught High School for 11 years before entering medicine at Queens University graduating in 1985. He practiced family practice for 25 years before focusing his practice on wound care. He is a past president and a founding board member of the Canadian Association of Wound Care. He is currently the president of the World Alliance for Wound and Lymphedema Care and Director of the Canadian Lymphedema Framework. Dr. Keast is an internationally recognized educator and has participated on many national and international advisory panels. ORCID: (https://orcid.org/0000‐0002‐3658‐2853)
**Professor Kazuo Kishi, Japan**	
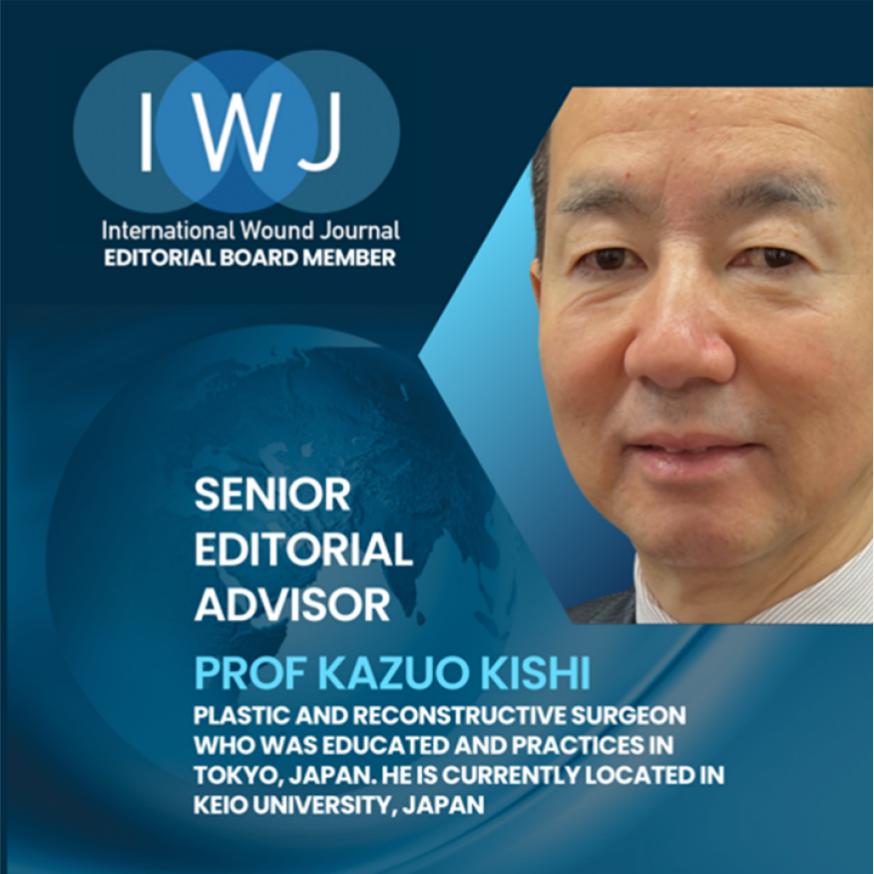	Professor Kishi is a plastic and reconstructive surgeon who was educated and practices in Tokyo, Japan. He is currently located in Keio University. Professor Kishi holds official position within 10 societies and is a member of 6 others. These cover the subject of plastic and reconstructive surgery, wound healing, burns, pressure ulcers and micro‐surgery. He is currently the president of the Japan Society of Plastic and Reconstructive Surgery. He serves on the editorial board of Wound Repair and Regeneration and is the Editor‐in‐Chief, International Journal of Surgical Wound Care. His clinical interests include wound healing, regenerative medicine, reconstructive surgery and surgical flaps. ORCID: (https://orcid.org/0000‐0002‐4298‐9828)
**Dr Stephan Landis, Canada**	
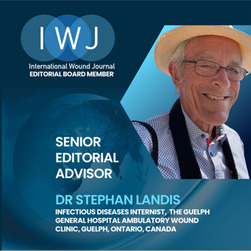	Dr. Stephan Landis is an infectious diseases internist, who has been involved in consultative wound care for the last 25 years. He is based at the Guelph General Hospital Ambulatory Wound Clinic, Guelph, Ontario, Canada.
**Professor Christine Moffatt, UK**	
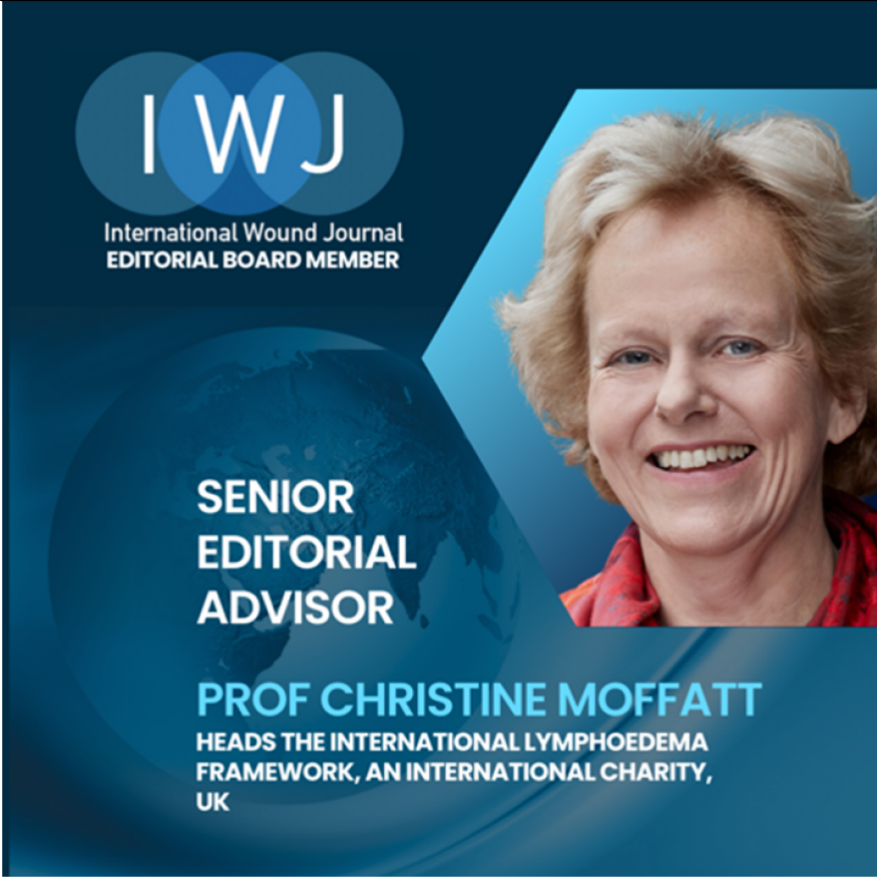	Professor Moffatt has been involved in wound healing for 30 years and lymphoedema for 20 years. She has undertaken extensive research in these fields using mixed research methods including running national and international clinical trials. She currently heads the International Lymphoedema Framework, an international charity, whose mission is to develop effective lymphoedema care throughout the world. She has presented and published internationally. Having edited the EWMA position document series and the International Lymphoedema Framework Best Practice Document (2006), Professor Moffatt participated on the NICE boards on leg ulcer management. Recently, she led the LIMPRINT study, an international epidemiology study with nine countries, that has defined the size and impact of the condition on health services. For services to healthcare, Christine was awarded a CBE in the 2006 Queens New Year's Honours List and made a life fellow of the Royal College of Nursing that same year. She has received six lifetime achievement awards between 2006 and 2015 in recognition of her work. ORCID: (https://orcid.org/0000‐0002‐2436‐0129)
**Professor Ricardo Roa, Chile**	
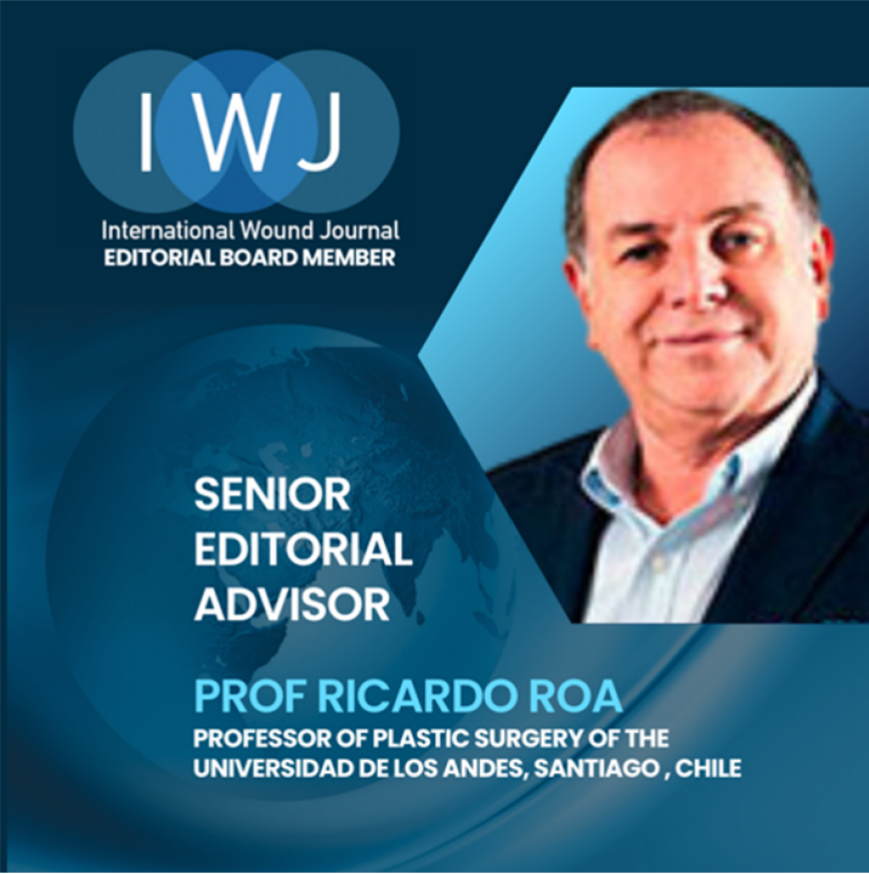	Professor Roa is a Plastic and Reconstructive Surgeon at the University of Concepcion, Chile. He has been the head of the Plastic Surgery and Burns Service for the Worker's Hospital, Santiago from 2011. He is currently the Professor of Plastic Surgery of the Universidad de los Andes, Santiago since 2023. Professor Roa is a founding member and past president of the Chilean Society of Burns and also a past president of the Latin American Federation of Burns. He is also currently the President of the International Society of Burn Injuries. He has published a pivotal book within Latin America—“Latin American Treaty on Wound Treatment”; over 50 scientific and clinical papers and nine book chapters. Professor Roa serves on several Editorial Boards and Committees.
**Professor Marco Romanelli, Italy**	
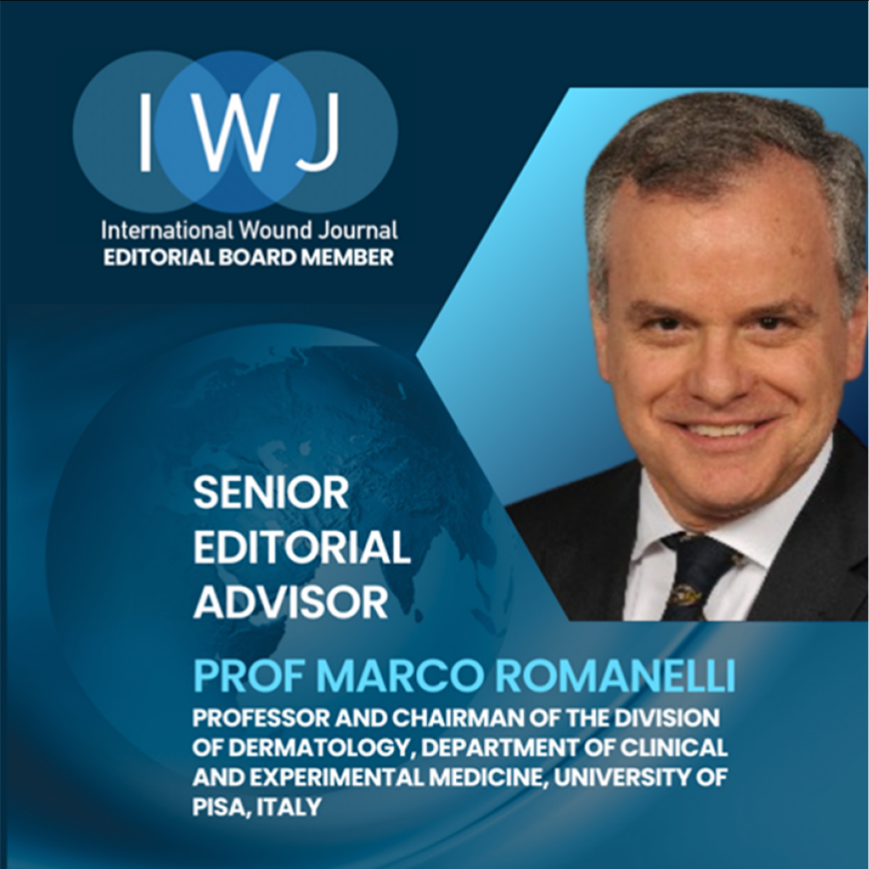	Dr. Romanelli is full professor and chairman of the Division of Dermatology, Department of Clinical and Experimental Medicine, University of Pisa, Italy. Dr. Romanelli is a graduate of the University of Pisa and received his MD and PhD in experimental dermatology from the medical faculty. He completed his residency in dermatology in Pisa and a fellowship at the Wound Healing Research Center, University of Miami, FL, USA. Subsequently, he specialized in medical hydrology. Since 2000, Dr. Romanelli has been a member of the Medical Faculty of the University of Pisa and adjunct associate professor at Department of Dermatology and Cutaneous Surgery, University of Miami Miller School of Medicine. Dr. Romanelli is a past president of the World Union of Wound Healing Societies. At national and international levels, Dr. Romanelli has served as member and chair of multiple grant review panels and on the editorial advisory boards of multiple journals. Dr. Romanelli has extensive clinical experience of managing patients with leg ulceration. He is responsible for the Wound Healing Research Unit inside the Santa Chiara Hospital at the University of Pisa. In 2006, he coordinated the European Pressure Ulcers prevalence project in Italy, which has put together data from over 25 national hospitals. ORCID: (https://orcid.org/0000‐0002‐4127‐0141)
**Professor Jose Contreras Ruiz, Mexico**	
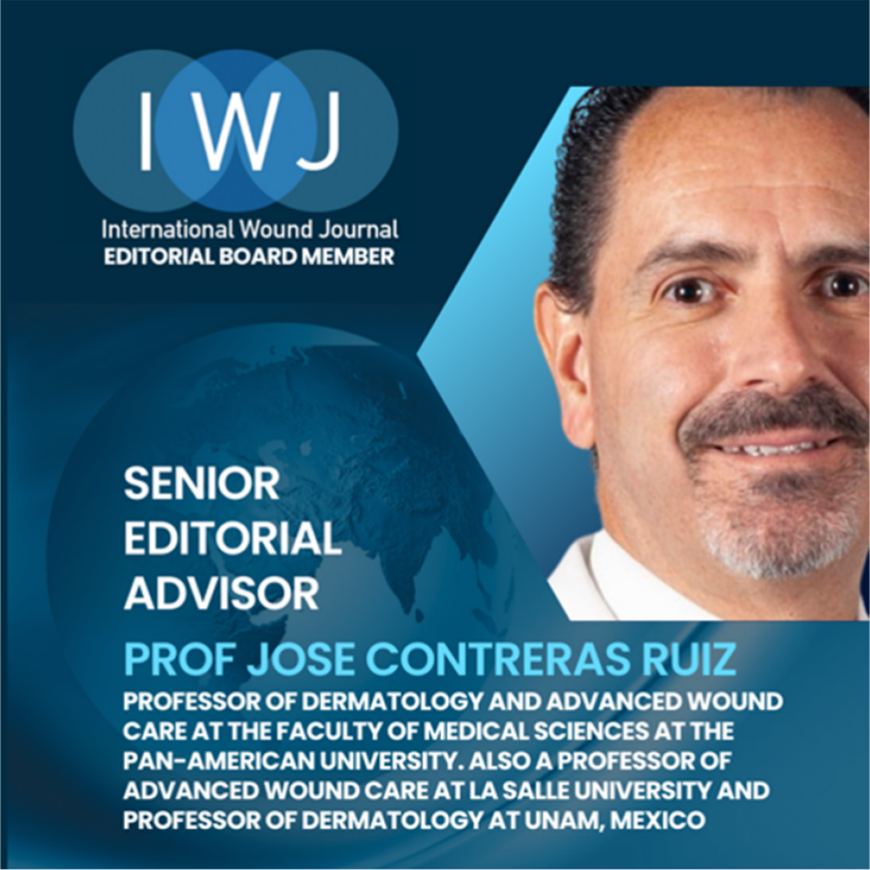	Dr. Contreras‐Ruiz graduated from La Salle University in México City, and spent his pre‐graduate internship at the University of Miami's Jackson Memorial Hospital in the United States where he had his first contact with wound care at the Department of Dermatology. He became a dermatologist at the “Dr. Manuel Gea Gonzalez” General Hospital, affiliated to the National University of Mexico (UNAM). While still a resident, he founded the Wound and Ostomy Care Centre within the Division of Dermatology (WOCC DermaGea), changing the way wounds were treated in México through evidence‐based practice thereafter. With Professor R. Gary Sibbald, MD, as his mentor, at the University of Toronto, Canada, he became the first International Wound Care Fellow. Back in Mexico, became the Head of the WOCC DermaGea and the founder and first president of AMCICHAC, the Mexican Association for Wound Care and Healing. He is a Professor of Dermatology and Advanced Wound Care at the Faculty of Medical Sciences at the Pan‐American University. He was also a Professor of Advanced Wound Care at La Salle University and Professor of Dermatology at UNAM. Dr. Contreras is a distinguished member of the National Academy of Medicine of Mexico, leads the CILAD (Ibero‐Latin‐American Dermatology College) chapter of wound care since 2016, and is considered a key opinion leader through Latin America. Dr. Contreras has published in both wound care and dermatology journals and published “Abordaje y Manejo de las Heridas”, the first wound care textbook written in Spanish. He currently resides in Los Cabos, México and is the Director at Centro Dermatológico Polanco – Los Cabos. ORCID: (https://orcid.org/0000‐0002‐5788‐9854)
**Professor Vijay Shukla, India**	
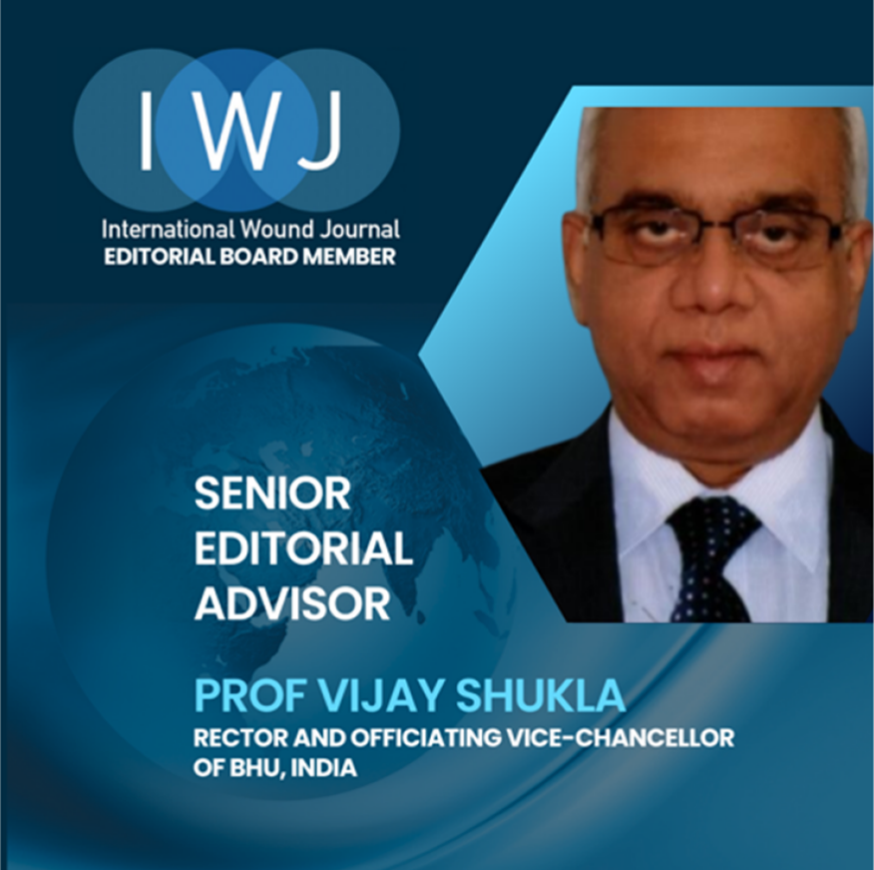	Prof. Vijay Kumar Shukla is an institution builder. During his term as Rector and Officiating Vice‐Chancellor of BHU, under his able leadership, several new initiatives and developments have taken place, which includes upgradation of Institute of Medical Sciences. During COVID 19, under the leadership, supervision and extensive monitoring of Prof. Vijay Kumar Shukla, the IMS‐BHU had served as Level 3 COVID Hospital with 469 beds (133 ICU and 336 HDU) for COVID patients having separate clinical investigation facility along with other facilities like dialysis, gynaecological and cardiac intervention, etc. More than 4500 COVID patients were admitted and treated in BHU. The hospital also extended healthcare services to the patients of Mucormycosis (Black Fungus). More than 250 patients were admitted and treated where 150 operations had been performed.
**Professor Geoff Sussman, Australia**	
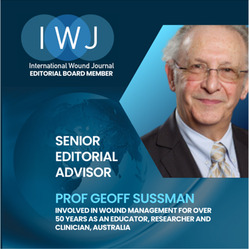	Geoff Sussman has been involved in wound management for over 50 years as an educator, researcher and clinician. He was responsible for the establishment of the Wound Foundation of Australia and was instrumental in the formation of the Australian Wound Management Association where he served as an executive board member for 8 years. He together with Professor Michael Stacey formed the World Union of Wound Healing Societies in 2000 and served on the executive board for 8 years. He was one of the first people in Australia to undertake clinical research in wound management and has over the years been involved as an investigator in many wound management studies; he published the results of a major wound healing outcome and health economic study done in Australian Nursing Homes. He trained with the leaders in the field in Scandinavia, Wales, United Kingdom, Europe and United States. He has been the innovator of teaching programmes and educational material including 46 book chapters. Geoff was awarded the Medal of the Order of Australia in the Queen's Birthday Honours in 2006. ORCID: (https://orcid.org/0009‐0001‐4819‐8728)
**Professor Ram Velamuri, USA**	
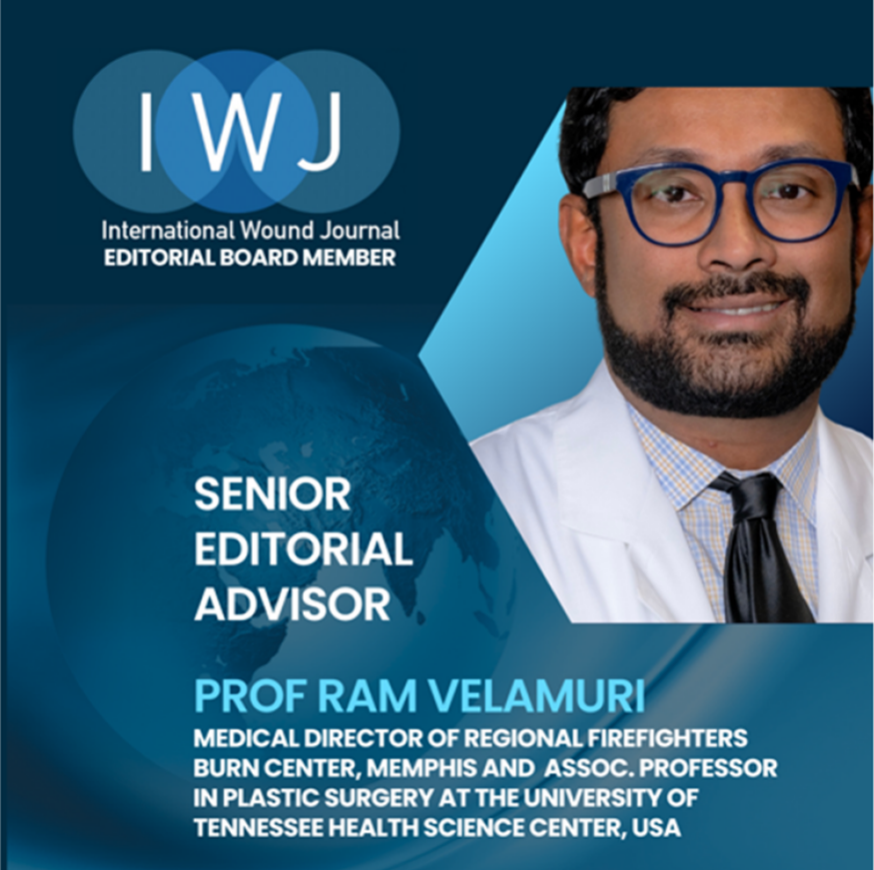	Dr. Velamuri is the Medical Director of Regional Firefighters Burn Center, Memphis. He is an associate professor in plastic surgery at the University of Tennessee Health Science Center. He is the current burn fellowship director. He serves on several regional and national committees and is the current chairman of the Global Health Committee of the American Burn Association. He earned his medical degree and completed a general surgery residency in India, and then moved to the United States to pursue training in plastic surgery. He completed residencies in general surgery and plastic and reconstructive surgery at Saint Louis University School of Medicine and a fellowship in burn and reconstructive plastic surgery at Johns Hopkins University.

Look out for a series of editorials in the coming month introducing our new board members by providing some background on their valued experiences. As the editorial team, we are excited to expand this group to help maintain the high‐quality standards of the International Wound Journal moving forward into our third decade of existence.

